# Effects of Gallic Acid Supplementation on Intestinal Function and Gut Microbial Community Structure in *Holothuria leucospilota*

**DOI:** 10.1155/anu/5350878

**Published:** 2026-07-24

**Authors:** Wenjie Pan, Hang Yuan, Chenchen Sun, Jianfeng Xu, Yi Zhang, Zhou Qin, Jingxuan Liang, Fanjiang Ou, Zepeng Zhang, Shuyang Wen, Chunhua Ren, Xiao Jiang, Peng Luo, Aifen Yan, Haipeng Qin, Chaoqun Hu, Ting Chen

**Affiliations:** ^1^ State Key Laboratory of Breeding Biotechnology and Sustainable Aquaculture, Laboratory of Tropical Marine Bio-resources and Ecology, Guangdong Provincial Key Laboratory of Applied Marine Biology, South China Sea Institute of Oceanology, Chinese Academy of Sciences, Guangzhou 510301, China, cas.cn; ^2^ University of Chinese Academy of Sciences, Beijing 100049, China, ucas.ac.cn; ^3^ State Key Laboratory of Mariculture Biobreeding and Sustainable Goods, Yellow Sea Fisheries Research Institute, Chinese Academy of Fishery Sciences, Qingdao 266071, China, cafs.ac.cn; ^4^ Guangdong Laboratory for Lingnan Modern Agriculture, College of Marine Sciences, South China Agricultural University, Guangzhou 510642, China, scau.edu.cn; ^5^ School of Medicine, Foshan University, Foshan 528225, China, fosu.edu.cn; ^6^ Agro-Tech Extension Center of Guangdong Province, Huizhou 516081, China

**Keywords:** aquaculture, gallic acid, microbiome, nutrition, sea cucumber

## Abstract

Gallic acid (GA) is a plant‐derived polyphenol with antioxidant and antimicrobial activities, yet its efficacy as a functional feed additive in sea cucumbers remains unclear. Here, we systematically evaluated graded dietary GA supplementation in the tropical sea cucumber *Holothuria leucospilota* by integrating growth performance, body‐wall nutritional composition, intestinal histomorphology, digestive and antioxidant enzyme activities, gut microbial community profiles, and intestinal transcriptomic responses. Sea cucumbers were randomly assigned to six dietary treatments containing 0, 200, 400, 800, 1600, or 3200 mg kg^−1^ GA and fed for 75 days. GA elicited a pronounced nonlinear, dose‐dependent response. The intermediate dose (GA2; 400 mg kg^−1^) produced a relatively favorable response under the present feeding conditions, increasing final body weight and increasing the accumulation of crude protein and structural‐protein‐associated amino acids in the body wall, while preserving brush‐border integrity and significantly enhancing α‐amylase, lipase, cellulase, and superoxide dismutase (SOD) activities. Transcriptomic profiling revealed clear divergence between GA‐treated groups and the control (Con), with GA2 characterized by upregulation of pathways associated with digestive hydrolysis, nutrient transport, lipid utilization, and redox/detoxification processes. Microbiome analyses showed progressive community restructuring with increasing GA, including reduced Proteobacteria and enrichment of Firmicutes/Bacilli at high doses, decreased α‐diversity, and pronounced genus‐level turnover. Correlation‐based integration further resolved two host–microbe interaction modules that linked microbial taxa either to nutrient assimilation programs or to intestinal barrier and immune‐response programs. Collectively, these findings support GA as a promising functional additive for *H. leucospilota* within a narrow optimal‐dose window, while highlighting potential risks of dysbiosis and intestinal injury at excessive inclusion levels.

## 1. Introduction

Gallic acid (GA) (3,4,5‐trihydroxybenzoic acid) is a naturally occurring plant phenolic compound commonly found in galls, oak bark, tea leaves, and a wide range of fruits [1]. GA has attracted considerable interest owing to its diverse bioactivities, including potent antioxidant and anti‐inflammatory properties, as well as reported antibacterial, antiviral, and anticancer effects [2]. Mechanistically, GA can alleviate oxidative stress by scavenging reactive free radicals and can modulate inflammatory signaling by suppressing proinflammatory mediators (e.g., cytokines and inflammation‐associated enzymes) [3]. These attributes have driven the extensive evaluation of GA as a natural functional additive in foods, pharmaceuticals, and animal feeds [4].

In aquaculture, the application of GA and other plant‐derived polyphenols as dietary supplements has received increasing attention as a strategy to enhance the growth performance and host health [5–7]. Evidence from studies in finfish and crustaceans suggests that appropriate GA supplementation can improve growth traits, feed utilization, and the overall physiological status. For example, dietary inclusion of 150–600 mg kg^−1^ GA in common carp has been associated with increased weight gain and feed efficiency, elevated digestive enzyme activities, strengthened antioxidant defenses, and enhanced immune responses under stress [8, 9]. Consistent benefits have also been reported for structurally related phenolics such as chlorogenic and caffeic acids, which have been linked to improved digestive function, favorable intestinal morphology, reinforcement of barrier integrity, and modulation of gut microbial communities in diverse aquatic species [10–12]. Collectively, these findings support the notion that plant polyphenols can promote aquatic animal health through coordinated effects on digestion, redox homeostasis, mucosal function, and the microbiome [13].

The tropical sea cucumber *Holothuria leucospilota* is a high‐value holothurian species with growing aquaculture potential [14, 15], yet its production remains constrained by several persistent bottlenecks, including slow growth, delayed maturation, low larval survival, and limited access to suitable feed resources [16, 17]. Improving growth efficiency and nutrient deposition is therefore a key priority for the sustainable expansion of *H. leucospilota* farming [18]. While functional additives such as probiotics, polysaccharides, and phytochemicals have been widely explored in fish aquaculture, evidence for plant‐based antioxidants, including GA, in sea cucumbers is still scarce [19]. This gap is particularly important given the distinctive digestive physiology of holothurians and their intimate gut microbial associations, which may shape responses to dietary polyphenols in ways that differ from those observed in vertebrates [20, 21]. Species‐specific assessment is thus required to evaluate the efficacy and to inform rational formulation.

Here, we systematically examined the effects of graded dietary GA supplementation on growth performance, body‐wall nutritional composition, intestinal digestive enzyme activities, gut histomorphology, microbial community structure, and host transcriptomic responses in *H. leucospilota*. By integrating phenotypic, biochemical, histological, microbiome, and transcriptome‐level readouts, we aimed to clarify the nutritional and gut‐modulatory roles of GA in an echinoderm model and to define an optimal inclusion level for practical applications. Our findings are expected to address a key knowledge gap in holothurian nutrition and provide an evidence base for the development of functional feeds that support healthy growth in tropical sea cucumber aquaculture.

## 2. Materials and Methods

### 2.1. Animals and Husbandry Conditions

Adult *H. leucospilota* was collected on July 30, 2025 by local divers from Daya Bay, Guangdong Province, southern China. Individuals were held at the Daya Bay experimental station for 3 days to allow gut evacuation (removal of ingested sand and debris) and then transported to an aquaculture facility in Maoming, Guangdong. Healthy animals without visible lesions and with uniform body size (initial wet weight: 50.0 ± 1.5 g, mean ± SD) were selected for the feeding trial.

Prior to the experiment, sea cucumbers were acclimated for 7 days in an indoor seawater recirculating system and fed a basal diet. Culture water consisted of sand‐filtered natural seawater with a salinity maintained at ~30. Water temperature followed ambient room conditions, dissolved oxygen was kept at ≥5 mg L^−1^, and the photoperiod was set to 12 h light/12 h dark. Continuous aeration was provided throughout the acclimation and experimental periods. Approximately one‐third of the water volume was exchanged every 2 days, and uneaten feed and feces were removed promptly to maintain the water quality.

### 2.2. Diet Preparation and Experimental Groups

Based on previous studies, a diet consisting of *Sargassum* powder and sea mud in a 1:4 ratio was formulated to serve as the control (Con) diet in the present study [22, 23]. Five experimental diets were formulated by supplementing the Con diet with graded levels of GA at 200 (GA1), 400 (GA2), 800 (GA3), 1600 (GA4), and 3200 (GA5) mg kg^−1^ diet, respectively (Table 1). *Sargassum* powder was purchased from Laizhou Haifu Feed Co., Ltd. (Shandong, China). GA (purity ≥ 98%, HPLC) was purchased from Macklin Co., Ltd. (Shanghai, China) and stored at 4°C, protected from light throughout the experiment. Sea mud was collected from an intertidal zone in Penglai, Shandong Province. Prior to use, it was sterilized by high‐temperature treatment and ground to pass through a 400‐mesh sieve. During feed preparation, GA was thoroughly mixed with the basal feed ingredients under light‐protected conditions at room temperature. The mixed diets were air‐dried at low temperature, immediately sealed in light‐proof bags after drying, and stored at 4°C in the dark until use so as to minimize oxidative degradation of the polyphenolic compound. The diet preparation method was adapted from studies on feed additives in sea cucumbers reported by Wang et al. [24] and Huang et al. [25], and the feasibility of the GA supplementation protocol has been validated in a similar study by Ghafarifarsani et al. [9].

**Table 1:** Diet formulation of different treatments (dry‐matter basis, %).

Ingredients	Composition (%)					
	Con	GA1	GA2	GA3	GA4	GA5
Sea mud	80	79.98	79.96	79.92	79.84	79.68
Sargasso meal	20	20	20	20	20	20
Gallic acid	0	0.02	0.04	0.08	0.16	0.32
Total	100	—	—	—	—	—
Nutrient index	Proximate composition (%)					
Crude protein^a^	4.28	—	—	—	—	—
Crude lipid^a^	0.3	—	—	—	—	—
Crude ash^a^	78.5	—	—	—	—	—
Moisture^a^	4.5	—	—	—	—	—

^a^Crude protein, crude lipid, ash, and moisture contents were measured value.

### 2.3. Experimental Design and Feeding Management

A completely randomized design was employed. After acclimation, sea cucumbers were randomly assigned to the six dietary treatments, with three replicate tanks per treatment and five individuals per tank (18 tanks in total; 200 L each). The feeding trial lasted for 75 days. Animals were fed once daily in the afternoon at 1%–2% of their wet body weight. Water quality conditions were maintained as described above, and temperature, salinity, and dissolved oxygen were monitored and recorded regularly.

### 2.4. Sample Collection and Growth Performance

At the end of the 75‐day trial, feeding was withheld for 24 h prior to the sampling. The final body weight was recorded for each individual. Sea cucumbers were anesthetized on ice and dissected immediately to collect the body wall and intestinal tissues. After rinsing, body‐wall samples were stored at −20°C for subsequent nutritional analyses. Intestinal tissues were snap‐frozen in liquid nitrogen and transferred to −80°C for transcriptomic analysis (*n* = 3), gut microbiome profiling (*n* = 4), and enzyme activity assays (*n* = 3). All procedures were conducted on ice or under chilled conditions to preserve the sample integrity.

### 2.5. Body‐Wall Nutritional Composition and Amino Acid Analysis

Body‐wall tissues stored at −20°C were dried at 65°C to constant weight and ground into a powder before analysis. The measured values were converted and expressed on a wet‐weight basis. Crude protein was determined by the Kjeldahl method, crude lipid by Soxhlet extraction, and ash by combustion in a muffle furnace. Amino acid composition and hydroxyproline (Hyp) content were determined after acid hydrolysis. Briefly, samples were hydrolyzed with 6 mol L^−1^ HCl under nitrogen at 110°C for 22 h, neutralized, diluted to volume, filtered, and analyzed using an automatic amino acid analyzer. Sixteen conventional amino acids and Hyp were quantified. Crude protein, crude lipid, ash, total amino acids, individual amino acids, and Hyp were expressed as g 100 g^−1^ wet weight. All procedures followed standard AOAC methods [26].

### 2.6. Intestinal Histology

Intestinal tissues fixed in 4% paraformaldehyde were dehydrated through a graded ethanol series, cleared in xylene, and embedded in paraffin. Serial sections (5 μm) were cut using a rotary microtome. Sections were deparaffinized, rehydrated, and stained with hematoxylin and eosin (H&E): hematoxylin for 5 min, bluing under running water, followed by eosin counterstaining for 2 min. After dehydration and clearing, sections were mounted with a neutral resin. Slides were examined under a light microscope (Nikon Eclipse E100). Digital images were acquired using an automated slide scanning system (Pannoramic MIDI) for morphological assessment.

### 2.7. Intestinal Digestive and Antioxidant Enzyme Activities

Fresh intestinal tissues were homogenized on ice in prechilled physiological saline at a ratio of 1:9 (w/v). Homogenates were centrifuged at 12,000×*g* for 10 min at 4°C, and the supernatants were collected as crude enzyme extracts. The total protein concentration was determined using the BCA method. Enzyme activities were measured using commercial kits (Nanjing Jiancheng Bioengineering Institute). The activities of amylase, lipase, pepsin, cellulase, superoxide dismutase (SOD), and catalase (CAT) were measured using commercial kits. One unit (U) of activity was defined as the amount of enzyme required to catalyze the formation of 1 μmol product min^−1^. Activities were normalized to the protein content and expressed as U mg^−1^ protein. In addition, malondialdehyde (MDA) content, an end product of lipid peroxidation, was determined in the same supernatant using a commercial kit (Nanjing Jiancheng Bioengineering Institute) based on the thiobarbituric acid (TBA) method according to the manufacturer’s instructions and was expressed as nmol mg^−1^ protein.

### 2.8. Intestinal Transcriptome Sequencing and Differential Expression Analysis

Based on the screening results of prior growth performance and physiological/biochemical indicators, four groups were selected for transcriptome analyses: Con, GA1, GA2, and GA5. Total RNA was extracted from intestinal tissues using a commercial kit (Omega Bio‐tek) according to the manufacturer’s protocol. RNA integrity was assessed by 1% agarose gel electrophoresis, and RNA concentration and purity were measured using a NanoDrop 2000 spectrophotometer. Libraries were prepared from qualified RNA using the V8 RNA‐seq Library Prep Kit (Vazyme) and sequenced on an Illumina Xplus platform in the paired‐end mode. Raw reads were quality‐filtered and adapter‐trimmed to obtain clean reads, which were mapped to the *H. leucospilota* reference genome (accession: PRJNA747844) [27]. Differential gene expression was evaluated using DESeq2 with thresholds of |log_2_ fold change| ≥ 1 and FDR < 0.05. KEGG pathway enrichment analysis was performed on differentially expressed genes (DEGs).

### 2.9. Gut Microbiome Profiling

Based on the screening results of prior growth performance and physiological/biochemical indicators, four groups were selected for intestinal microbiota analyses: Con, GA1, GA2, and GA5. Microbial genomic DNA was extracted from intestinal samples using the HiPure Soil DNA Mini Kit (TIANGEN), following the manufacturer’s instructions. The full‐length 16S rRNA gene was amplified by PCR. Amplicons were gel‐purified and quantified prior to library construction using the SQK‐LSK114 kit (Oxford Nanopore Technologies [ONT]). Library quality was assessed before sequencing on an R10.4.1 flow cell using a PromethION platform. Raw signals were basecalled with Guppy (v6.4.6). Adapters were removed with Porechop, and reads were quality‐filtered with NanoFilt (*Q* ≥ 7; length ≥ 1000 bp). High‐quality reads were aligned to the SILVA 16S rRNA database (v138) using the LAST to generate a feature table. Features with relative abundance ≤ 1% in each sample were removed, and the dataset was rarefied to the minimum sequencing depth for downstream analyses of diversity and community composition.

### 2.10. Statistical Analysis

Statistical analysis was performed using GraphPad Prism 7.0. All data are presented as the mean ± SD unless otherwise stated. Statistical differences were assessed using one‐way ANOVA, followed by Tukey’s multiple comparisons test.

## 3. Results

### 3.1. Growth Performance and Body‐Wall Nutritional Composition

**Figure 1:**
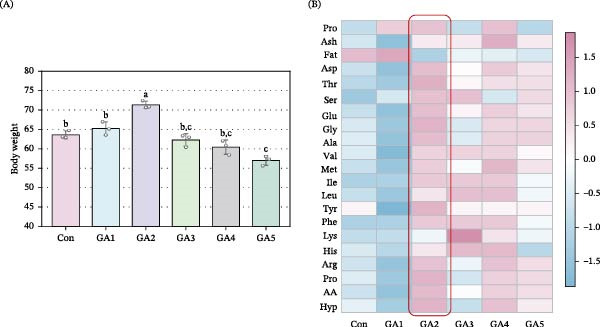
Effects of graded dietary gallic acid (GA) supplementation on final body weight and body‐wall nutritional composition in *H. leucospilota*. (A) Final body weight (g) of sea cucumbers after the 75‐day feeding trial across dietary treatments (Con, GA1–GA5). Bars show mean ± SD, and points represent tank‐level means (*n* = 3 tanks per treatment). Different letters above bars indicate significant differences among groups. (B) Heatmap showing body‐wall nutritional composition across treatments. Values are *Z*‐score standardized by row to depict relative changes among groups.

Graded dietary GA supplementation significantly affected the final body weight of *H. leucospilota* in a nonlinear, dose‐dependent manner (Figure 1A). Relative to the Con, the low‐dose treatment (GA1) showed only a slight increase in body weight and did not differ significantly from that of Con, indicating a limited growth‐promoting effect at this level. In contrast, the intermediate dose (GA2) yielded the highest final body weight and was significantly greater than that of all other treatments, indicating that GA2 was associated with the highest growth performance under the present conditions (Table 2). With further increases in GA, final body weight declined to intermediate levels in GA3 and GA4, partially overlapping with both Con/GA1 and GA2 and showing no sustained benefit. The highest‐dose group (GA5) exhibited the lowest body weight and was significantly lower than those in the Con, GA1, and GA2 groups.

**Table 2:** Survival and growth performance of sea cucumbers under different dietary GA treatments.

Treatment	Initial	Final	Survival (%)	Initial body weight (g)	Final body weight (g)	WGR (%)	SGR (% d^−1^)
Con	15	15	100	49.78 ± 0.50^a^	63.59 ± 3.89^b^	27.77 ± 8.22^b^	0.434 ± 0.119^b^
GA1	15	15	100	50.47 ± 0.41^a^	65.23 ± 3.00^ab^	29.26 ± 5.93^b^	0.457 ± 0.081^b^
GA2	15	15	100	49.86 ± 0.29^a^	71.31 ± 2.63^a^	43.03 ± 5.37^a^	0.638 ± 0.067^a^
GA3	15	15	100	50.34 ± 0.30^a^	62.26 ± 2.23^bc^	23.68 ± 4.52^bc^	0.378 ± 0.065^bc^
GA4	15	15	100	49.72 ± 0.31^a^	60.43 ± 2.64^c^	21.53 ± 5.18^c^	0.347 ± 0.077^c^
GA5	15	15	100	49.99 ± 0.27^a^	57.00 ± 1.92^d^	14.03 ± 3.78^d^	0.234 ± 0.060^d^

*Note:* Values with different small letter superscripts mean significant difference (*p* < 0.05).

Body‐wall nutritional composition was also altered by GA supplementation (Figure 1B). The crude protein content was higher in GA1, GA2, and GA4 (7.45%, 7.58%, and 7.59%, respectively) than in Con (6.36%). The total amino acid content peaked in GA2 (6.31%), representing an 18.4% increase over Con (5.33%). The crude lipid content remained relatively stable among treatments (10.22%–10.76%), and the ash content varied only slightly (3.0%–3.3%). Notably, amino acids associated with structural proteins in the body wall, including glutamic acid, glycine, proline, and Hyp, reached relatively high or peak levels in GA2. Essential amino acid levels varied only moderately among GA‐supplemented groups, and their proportion within total amino acids remained relatively stable (25%–28%).

### 3.2. Intestinal Histology and Enzyme Activities

**Figure 2:**
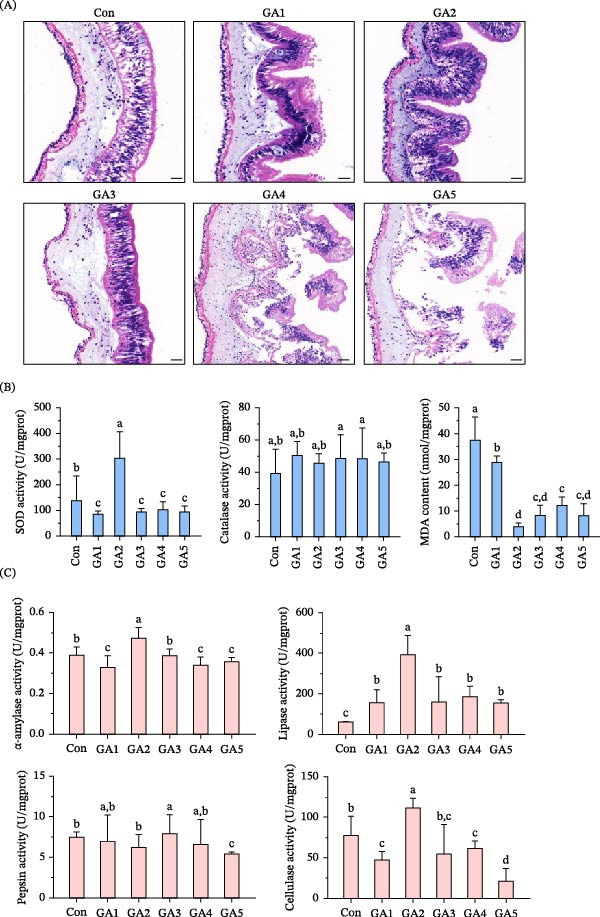
Intestinal histomorphology and enzyme activities in *H. leucospilota* under graded dietary gallic acid (GA) supplementation. (A) Representative hematoxylin and eosin (H&E)‐stained intestinal sections from sea cucumbers in the control (Con) and GA‐supplemented groups (GA1–GA5) after the 75‐day feeding trial. Images were observed and captured under 20× magnification. Black scale bars represent 50 μm. (B) Antioxidant‐related indicators in intestinal tissue, including superoxide dismutase (SOD), catalase (CAT), and malondialdehyde (MDA). SOD and CAT activities are expressed as U mg^−1^ protein, and MDA content is expressed as nmol mg^−1^. (C) Digestive enzyme activities in intestinal tissue, including α‐amylase, lipase, pepsin, and cellulase, expressed as U mg^−1^ protein. For B and C, bars show mean ± SD. Different letters above bars indicate significant differences among treatments (*p* < 0.05; one‐way ANOVA followed by Tukey’s multiple‐comparison test).

Histological examination indicated that the intestinal architecture in Con was intact, with a regular brush‐border morphology (Figure 2A). In GA1 and GA2, the brush border appeared more developed, and the epithelium remained continuous, whereas clear brush‐border damage was observed at higher inclusion levels (GA4 and GA5). Consistent with these morphological patterns, marked changes were detected in digestive and antioxidant enzyme activities (Figure 2B,C). Among antioxidant enzymes, SOD activity was highest in GA2 and was significantly greater than that in the other groups. The MDA content showed an opposite trend to SOD activity, with the lowest level observed in GA2. This indicated that the enhanced SOD activity in GA2 was not accompanied by increased lipid peroxidation damage. For digestive enzymes, GA2 exhibited peak activities of α‐amylase, lipase, and cellulase. Pepsin activity was relatively higher in GA3 but showed a declining trend in GA5. Collectively, these results suggest that a moderate GA level (particularly GA2) supported intestinal structural integrity, enhanced antioxidant enzyme responses, reduced lipid peroxidation, and improved digestive enzyme activities.

### 3.3. Transcriptome‐Wide Responses and DEGs

**Figure 3:**
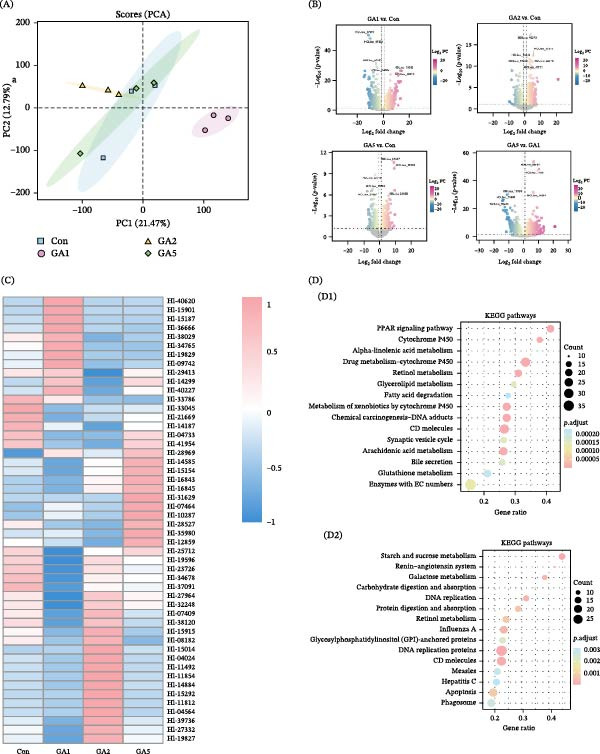
Transcriptomic responses of the intestine to graded dietary gallic acid (GA) supplementation in *H. leucospilota*. (A) Principal coordinates analysis (PCoA) based on intestinal transcriptomic profiles, showing separation of the control (Con) and GA‐treated groups (GA1, GA2, and GA5). Each point represents one biological replicate, and shaded ellipses indicate the 95% confidence interval for each group. (B) Volcano plots showing differentially expressed genes (DEGs) for representative pairwise comparisons (GA1 vs. Con, GA2 vs. Con, GA5 vs. Con, and GA5 vs. GA1). (C) Heatmap of the top 50 DEGs (*Z*‐score–standardized expression) across Con, GA1, GA2, and GA5. (D) KEGG pathway enrichment analysis of DEGs. Bubble plots show significantly enriched pathways for upregulated (D1) and downregulated (D2) DEGs, with the *x*‐axis representing the gene ratio.

Principal coordinates analysis (PCoA) based on intestinal transcriptomic profiles revealed clear separation between GA‐treated groups and Con, indicating treatment‐associated shifts in global gene expression patterns (Figure 3A and Supporting Information 1 Tables S1 and S2). Replicates within each group clustered within their respective confidence ellipses, supporting dataset consistency. Among the GA treatments, GA1 showed the strongest divergence from Con, followed by GA2. Differential expression analysis corroborated these patterns: relative to Con, GA1, GA2, and GA5 yielded 2206, 983, and 633 DEGs, respectively (Figure 3B and Supporting Information 1 Table S3). Pairwise comparisons among treatments further indicated pronounced transcriptional reprograming between GA2 and GA1 (2661 DEGs) and between GA5 and GA1 (2073 DEGs), whereas GA5 versus GA2 showed fewer DEGs (822).

### 3.4. Expression Patterns of the DEGs

Swiss‐Prot annotation and functional clustering of the top 50 DEGs (*Z*‐score–standardized expression) indicated that the dominant responses were concentrated in modules related to digestive hydrolysis, nutrient absorption and transport, detoxification, and antioxidant defense (Figure 3C and Supporting Information 1 Table S4). Genes involved in digestion and nutrient hydrolysis were most prominently upregulated in GA2, including α‐amylase (*Hl-15292*), maltase (*Hl-15915*), multiple serine/trypsin‐like proteases (*e.g*., *Hl-11812* and *Hl-11854*), and a pancreatic lipase‐related protein (*Hl-14884*). Genes associated with lipid transport and metabolism, such as apolipoprotein B (APOB) (*Hl-15014*) and fatty acid‐binding protein 2 (*Hl-07409*), also exhibited their highest expression in GA2. In contrast, transcripts linked to protein turnover showed dose‐dependent divergence: cathepsin L (*Hl-19829*) was more highly expressed in GA1, whereas cathepsin L1 (*Hl-19827*) was markedly elevated in GA2. Notably, the expression of mucosal barrier‐related genes was more pronounced in GA1, with mucin MUC5B (*Hl-40620*) and DMBT1 (*Hl-15187*) reaching their highest expression levels, suggesting that low‐dose GA may preferentially support mucosal barrier maintenance or reinforcement.

### 3.5. KEGG and GO Enrichment Analyses

KEGG and GO enrichment analyses collectively indicated that GA supplementation substantially reshaped intestinal metabolic and stress‐response regulatory networks in *H. leucospilota* (Figure 3D and Supporting Information 1 Table S5). KEGG enrichment analysis showed that upregulated DEGs were primarily enriched in pathways associated with lipid utilization and transport and redox/detoxification functions, including PPAR signaling, fatty acid degradation, bile secretion, glutathione metabolism, and cytochrome P450‐mediated xenobiotic metabolism. GO results supported this pattern: upregulated genes were significantly enriched in biological processes such as dephosphorylation, oxidation–reduction, and transmembrane transport, and in molecular functions including heme/iron ion binding and oxidoreductase activity, consistent with broad enhancement of membrane transport capacity and redox homeostasis (Supporting Information 2 Figure S1 and S2). Conversely, downregulated DEGs were significantly enriched in KEGG pathways related to nutrient metabolism and core cellular processes, including starch and sucrose metabolism, carbohydrate digestion and absorption, protein digestion and absorption, as well as DNA replication, apoptosis, and phagosome pathways. GO enrichment similarly linked downregulated genes to DNA replication initiation, microtubule‐related processes, nuclear functions, carbohydrate metabolic processes, proteolysis, and ATP binding, suggesting suppression of cell proliferation‐associated programs together with reduced activity in subsets of basal metabolic functions.

### 3.6. Gut Microbial α‐Diversity and Venn Diagram Analysis

Alpha‐diversity metrics indicated marked GA‐associated changes in intestinal microbial richness and evenness (Figure 4A and Supporting Information 1 Table S6). Across all samples, the number of observed features ranged from 85 to 732. The Con group exhibited the highest Shannon diversity (6.330 ± 0.171), and diversity remained relatively high in the low‐dose group (GA1; 5.442 ± 0.211). In contrast, Shannon diversity decreased substantially under moderate and high GA exposure, with significantly lower values in GA2 (3.780 ± 0.960) and GA5 (2.954 ± 0.803) compared with those in Con.

Figure 4Dietary gallic acid (GA) reshapes gut microbial diversity and community composition in *H. leucospilota*. (A) Alpha‐diversity indices of the intestinal microbiome across treatments, including ACE, Chao1, Shannon, and Simpson. Boxplots show the median (center line), interquartile range (box), and range (whiskers), with points representing individual samples. (B) Venn diagrams showing shared and unique OTUs among groups. The upper panel compares the pooled GA treatments (GA) versus the control (Con), and the lower panel shows overlap among Con, GA1, GA2, and GA5. (C) Phylum‐level taxonomic composition of the intestinal microbiome across treatments. (D) Class‐level taxonomic composition of the intestinal microbiome across treatments. (E) Heatmap of genus‐level relative abundances across samples. Columns represent individual samples grouped by treatment and rows represent genera.

**Figure:**
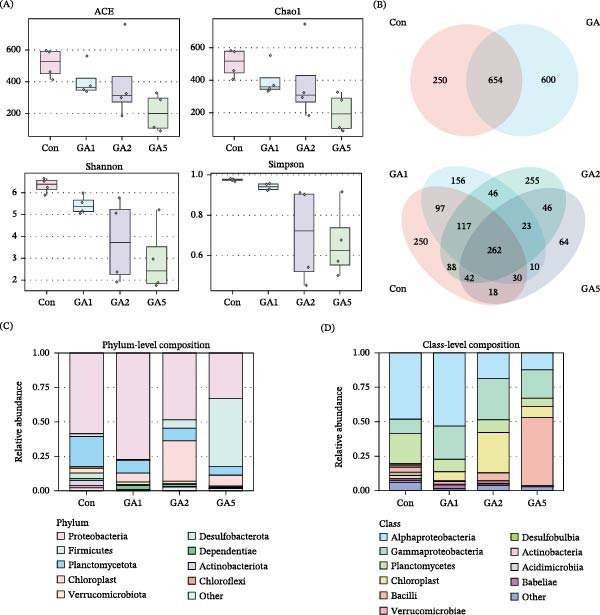

**Figure:**
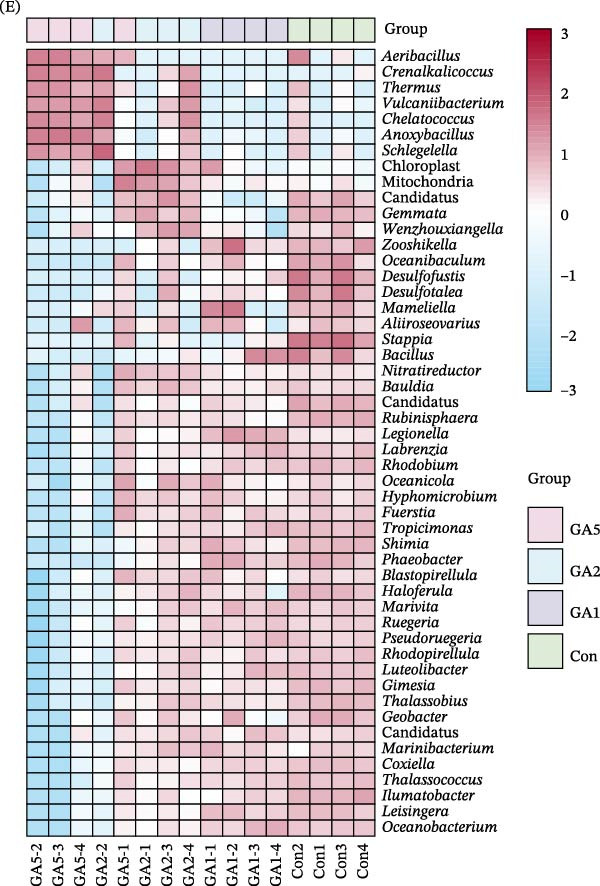

Venn diagram analysis further resolved shared versus treatment‐specific OTUs (Figure 4B). Among Con, GA1, GA2, and GA5, a core set of 262 OTUs was shared (17.42% of the union). The number of unique OTUs differed across treatments, with GA2 displaying the highest number of group‐specific OTUs (255). When all GA treatments were pooled (GA) and compared against Con, 654 OTUs were shared (43.48%); the GA pool and Con contained 600 and 250 unique OTUs, respectively, highlighting a pronounced restructuring of the intestinal community under GA supplementation.

### 3.7. Community Shifts at the Phylum and Class Levels

At the phylum level, Proteobacteria dominated all groups, yet its relative abundance declined with increasing GA dose, decreasing from 77.33% in GA1 to 32.93% in GA5 (Figure 4C and Supporting Information 1 Table S7). In parallel, Firmicutes exhibited a strong dose‐dependent enrichment, increasing from 1.85% in Con to 49.58% in GA5. Planctomycetota was comparatively abundant in Con (21.97%) but decreased to less than 10% in all GA‐treated groups.

Class‐level profiles mirrored these patterns (Figure 4D and Supporting Information 1 Table S8). Con was characterized primarily by Alphaproteobacteria (48.21%) and Planctomycetes (21.83%). In GA1, Alphaproteobacteria remained high (53.31%), while Gammaproteobacteria increased to 24.01%. In GA2, Gammaproteobacteria further increased (30.08%), accompanied by a reduction in Alphaproteobacteria (18.53%). Notably, the high‐dose treatment (GA5) displayed a pronounced community turnover, with Bacilli (Firmicutes) rising sharply from 1.43% in Con to 49.46%, consistent with the strong Firmicutes enrichment observed at the phylum level.

### 3.8. Dominant Taxa Turnover at the Genus Level

Genus‐level composition revealed a clear replacement of dominant lineages across treatments (Figure 4E and Supporting Information 1 Table S9). The Con microbiota was dominated by typical marine Rhodobacteraceae‐associated genera, with *Ruegeria* (10.42% ± 0.84%), *Labrenzia* (10.45% ± 2.12%), and *Pseudoruegeria* (8.23% ± 0.82%) forming the dominant backbone. These taxa remained comparatively abundant under low‐dose GA (GA1).

In the moderate‐dose group (GA2), *Vulcaniibacterium* increased markedly, and *Anoxybacillus* showed an upward trend; concomitantly, Rhodobacteraceae‐associated genera declined (*e.g*., *Ruegeria* to 3.45% ± 2.06% and *Labrenzia* to 2.46% ± 0.94%). The most substantial genus‐level shift occurred in the high‐dose group (GA5), where *Anoxybacillus* expanded dramatically and became the dominant genus (48.78% ± 16.54% vs. 0.65% ± 0.60% in Con), while *Vulcaniibacterium* remained relatively high (17.56% ± 6.00%). In parallel, genera that dominated Con declined to low abundance, indicating a high‐dose GA‐associated restructuring toward a Firmicutes/Bacilli‐centered community.

### 3.9. Correlation‐Based Host Transcriptome–Microbiome Associations

**Figure 5:**
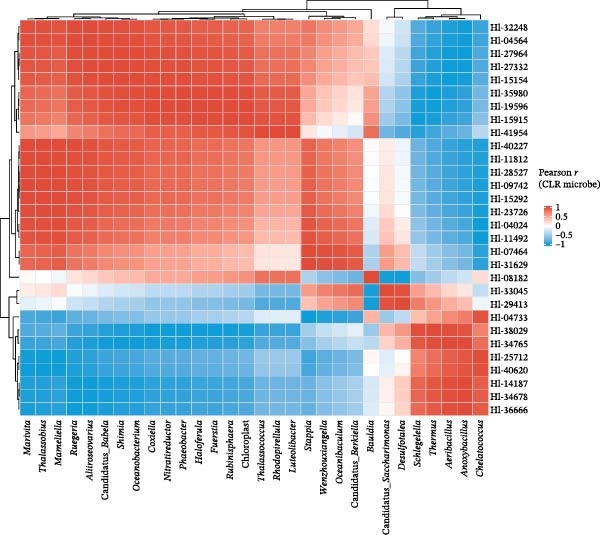
Integrated host transcriptome–microbiome associations in the intestine of *H. leucospilota* under dietary gallic acid (GA) supplementation. Heatmap showing Pearson correlations between microbial taxa and host genes. Rows and columns were clustered by hierarchical clustering. Red and blue indicate positive and negative correlations, respectively.

Correlation‐based integration of intestinal transcriptomes with microbial community profiles (Pearson correlation with bidirectional hierarchical clustering) resolved two antagonistic modules linking microbial taxa to host functional programs (Figure 5A). Module I was centered on marine heterotrophic/symbiotic lineages, including *Thalassobius*, *Mameliella*, and *Ruegeria*, and showed significant positive associations with a suite of host genes involved in nutrient acquisition, digestive processing, and transport. Representative transcripts included aminopeptidase N (*Hl-19596*), maltase (*Hl-15915*), and vitellogenin‐1 (*Hl-15154*), collectively suggesting coordinated upregulation of digestion/absorption capacity in the presence of these taxa.

By contrast, Module II was dominated by *Anoxybacillus*, *Bacillus*, and *Aeribacillus* and correlated positively with genes linked to mucosal barrier maintenance, innate immune activity, and oxidative stress responses, including mucin MUC5B (*Hl-40620*), dual oxidase (*Hl-14187*), and CAT (*Hl-25712*). Importantly, Module II taxa and gene sets were broadly negatively correlated with Module I, supporting a trade‐off‐like pattern in which GA‐associated microbial restructuring was linked to either a nutrient‐processing program (Module I) or a barrier/immune‐oxidative defense program (Module II).

## 4. Discussion

Our results indicate that dietary GA affected growth performance and intestinal health in *H. leucospilota* in a dose‐dependent but nonlinear manner [28]. Among the tested inclusion levels, the intermediate dose (GA2) produced the most favorable integrated outcome, as reflected by a significant increase in final body weight together with elevated body‐wall crude protein and increased accumulation of structural‐protein‐associated amino acids. These changes suggest that an appropriate GA level may improve body‐wall protein accumulation and growth under the present feeding conditions [2, 29]. In contrast, higher GA inclusion (GA4/GA5) was accompanied by growth suppression, intestinal tissue injury, and a destabilized microbial profile, collectively consistent with a biphasic “low‐benefit/high‐cost” pattern. Such nonlinear responses have been observed for several functional additives in holothurian culture, implying that efficacy often depends on maintaining supplementation within a biologically effective concentration range.

Histological and enzymatic evidence directly supports the phenotypic divergence observed among treatments [30]. In GA1 and GA2, the intestinal mucosa displayed well‐defined folds and preserved architecture, indicating maintenance of the absorptive surface area and epithelial barrier integrity. Consistent with this pattern, GA2 exhibited peak activities of key digestive enzymes (α‐amylase, lipase, and cellulase) together with the highest SOD activity, pointing to a coordinated enhancement of nutrient hydrolysis capacity and antioxidant buffering. Notably, the MDA content in the GA2 group was significantly lower than that in the Con and other treatment groups, indicating that the elevated antioxidant enzyme activity was not accompanied by increased lipid peroxidation damage. This opposite trend between SOD activity and MDA content further supports the interpretation that GA2 enhanced the intestinal antioxidant defense capacity in a physiological manner rather than representing a compensatory response to oxidative injury. Similar coupled improvements in digestive and antioxidant enzyme activities have been reported in sea cucumbers receiving functional dietary interventions [31, 32]. For instance, supplementing with compound lactic acid bacteria has been shown to elevate intestinal digestive enzymes and SOD, while dietary glutamine supplementation increases protease/lipase activities and antioxidant enzymes (SOD and CAT), alongside improving gut morphology [6, 33]. Together, these observations suggest that the beneficial effect of GA2 may be associated with improved digestive enzyme activity and antioxidant enzyme responses. By contrast, the mucosal disorganization observed at high GA doses, together with the concomitant decline in digestive enzyme activities, is more consistent with disruption of intestinal homeostasis, leading to reduced digestive efficiency and, ultimately, constrained growth [34].

At the molecular level, the transcriptome revealed a clear dose‐specific regulatory landscape. PCoA indicated robust separation between GA treatments and the Con. Specifically, the number of DEGs did not scale monotonically with dose, with GA1 yielding the largest DEG set relative to that of the Con. This pattern suggests that distinct GA doses may activate qualitatively different physiological programs rather than progressively amplifying a single response. At the transcriptomic level, GA2 was characterized by a coordinated upregulation of genes involved in nutrient digestion and absorption, including digestive hydrolytic enzymes and nutrient transport factors, accompanied by a reinforced lipid assimilation and trafficking module, as evidenced by elevated expression of key lipid‐handling genes such as APOB and fatty acid–binding protein 2 (FABP2). This expression pattern is consistent with enhanced intestinal lipid transport and metabolism. FABP2 is a central mediator of long‐chain fatty‐acid transport within enterocytes and influences their metabolic partitioning [35, 36], whereas APOB is an essential structural component required for lipoprotein assembly and lipid export, analogous to the chylomicron biogenesis machinery described in vertebrates, thereby reflecting an enhanced capacity to convert dietary lipids into bioavailable nutrients [37]. These molecular features align with the elevated digestive enzyme activities and improved growth performance observed in GA2, supporting a functionally coherent linkage between transcriptional reprograming and organismal outcomes [38, 39]. Notably, enrichment of the PPAR signaling pathway suggests that the strengthened lipid metabolism observed here is unlikely to be a purely passive consequence of the increased substrate flux. Instead, it is consistent with an active metabolic remodeling program governed by a lipid‐sensing transcriptional network. As central regulatory nodes, PPAR family members typically integrate fatty acid cues with redox status and inflammatory signals, thereby coordinately regulating lipid absorption, fatty acid oxidation, lipoprotein metabolism, and the magnitude of immune responses. Through this integrative Con, PPAR signaling can help maintain a dynamic balance between efficient energy acquisition and the inflammatory costs associated with heightened metabolic activity [40]. In contrast, GA1 displayed a transcriptomic signature biased toward mucosal barrier maintenance, with a significant upregulation of mucin‐related genes. As core components of the intestinal physical and chemical barrier, mucins not only limit pathogen adhesion and invasion but also provide adhesion niches and substrates that can help stabilize commensal communities [41]. The GA1 expression profile therefore suggests a prioritization of resource allocation toward barrier renewal and immune homeostasis, which may help explain why GA1 yielded a comparatively large DEG set but conferred limited growth gains. At the highest GA level (GA5), stress‐responsive transcripts were strongly induced, including ferritin, consistent with heightened oxidative stress and perturbation of iron homeostasis. Ferritin is not only the principal intracellular iron‐storage protein that limits the pool of labile iron capable of catalyzing reactive oxygen species (ROS) formation but also contributes directly to redox buffering. In particular, the H subunit possesses ferroxidase activity that oxidizes Fe(II) to Fe(III), thereby facilitating safe iron sequestration and mitigating iron‐driven oxidative damage [42, 43]. Its upregulation is thus consistent with the intestinal damage and growth suppression observed in GA5, supporting a shift toward a defense‐ and repair‐dominant physiological state under excessive GA exposure [44].

Gut microbiome profiling indicated that GA supplementation markedly reshaped both microbial diversity and community structure in the intestine [45]. Although α‐diversity showed an overall decline across GA‐treated groups, it is important to note that reduced diversity alone does not directly imply compromised health in cultured animals [46, 47]. Rather, the more relevant determinant is whether the intestinal community maintains a balanced structure and functional integrity. In our dataset, the high‐dose treatments were characterized by a pronounced reduction in α‐diversity together with an overt turnover of dominant taxa, most notably a strong expansion of Firmicutes and Bacilli. Crucially, these microbial shifts coincided with intestinal histological injury, suggesting that excessive GA may destabilize the gut ecosystem and undermine intestinal homeostasis [48]. Accordingly, microecological disturbance in this context is best diagnosed by integrating host physiological readouts: abrupt community restructuring becomes biologically meaningful when it is coupled to clear evidence of tissue damage and functional suppression, a pattern that was most evident in GA4/GA5 [49]. Because microbiome profiling mainly reflects changes in the community composition rather than direct microbial metabolic activity, the functional consequences of these community shifts remain inferential. Nevertheless, recent studies using botanical gastrointestinal intervention models have linked microbiota remodeling with inflammatory regulation, oxidative damage mitigation, intestinal barrier restoration, and increased SCFA production, suggesting that SCFAs and related microbial metabolites may be candidate intermediates connecting GA‐associated microbial shifts with host intestinal responses [50, 51].

Notably, correlation‐based integration of transcriptomic and microbial profiles provides clues that GA‐associated microbial restructuring was linked to distinct host intestinal transcriptional programs [52, 53]. Marine heterotrophic taxa such as *Thalassobius* and *Mameliella* were positively associated with host genes involved in digestion/absorption and lipid transport. These lineages, largely affiliated with Rhodobacteraceae and related marine clades, are recognized for efficient utilization of dissolved organic matter and have been reported in aquaculture settings to suppress potential pathogens, potentially through antagonistic metabolite production and/or competitive exclusion [54]. Ecologically, they therefore align more closely with homeostasis‐promoting taxa or probiotic‐like components of the intestinal community [54]. By contrast, Bacilli‐associated taxa typified by *Anoxybacillus* showed positive correlations with gene sets linked to mucosal barrier maintenance, antioxidant capacity, and innate immune defense while exhibiting broadly negative associations with the nutrient‐assimilation module. This pattern does not negate the probiotic utility of Bacilli in aquaculture; rather, it underscores the importance of the ecological context. In the high‐dose treatments, Bacilli taxa (*e.g*., *Anoxybacillus*) rapidly rose to dominance via a bloom‐like community replacement. This pattern is more consistent with disturbance‐driven opportunistic expansion than with benign optimization of the intestinal ecosystem. Opportunistic blooms typically occur after perturbation disrupts an originally complex and diverse community. Such perturbation may create ecological niches that allow stress‐tolerant or fast‐growing Bacilli to expand rapidly [55, 56]. Importantly, although certain Bacilli strains are widely used as probiotics and can enhance host immunity and gut health under appropriate conditions, their dominance under a disturbed regime may instead reflect ecological imbalance rather than improved community function [52, 53].

Together, this antagonistic organization supports a trade‐off‐like balance between a nutrient‐assimilation–dominant state and a defense/repair–dominant state [28, 34]. Within this framework, an appropriate GA level (GA2) may preserve epithelial integrity while promoting a microbiome configuration and host transcriptional program that jointly favor nutrient assimilation, thereby supporting growth [39]. Conversely, excessive GA (GA4/GA5) may impose stronger antimicrobial selection and/or heightened physiological stress, destabilizing the microbial ecosystem and shifting host priorities toward defense and repair, ultimately culminating in intestinal injury and growth suppression. Collectively, this integrative model reconciles the dose‐dependent patterns observed across phenotypes, histology, enzyme activities, transcriptomics, and microbiome composition and underscores the importance of optimizing GA inclusion to maximize benefits while avoiding high‐dose costs in *H. leucospilota* culture.

## 5. Conclusion

In conclusion, by integrating growth performance with intestinal histology, digestive physiology, host transcriptional regulation, and gut microbial ecology, our study demonstrates that GA may have potential as a functional feed additive for *H. leucospilota*, but its efficacy is strongly dose‐dependent. The intermediate inclusion level (GA2, 400 mg kg^−1^) appeared to be the most suitable level under the present feeding conditions, yielding the most consistent improvements in growth and body‐wall nutritional quality, whereas higher doses were associated with an increased risk of intestinal dysbiosis and mucosal injury. Future work should prioritize the evaluation of the long‐term safety and stability of GA2 supplementation, validate its effectiveness across developmental stages and farming practices, and further clarify how GA interacts with the gut microbiome and host intestinal metabolism. Collectively, these efforts will strengthen the evidence base for developing functional feeding strategies for sea cucumber aquaculture.

## Author Contributions

**Wenjie Pan:** writing – original draft, writing – review and editing, methodology, formal analysis, software, data curation, validation. **Hang Yuan:** methodology, investigation, formal analysis, data curation, validation. **Chenchen Sun, Jianfeng Xu, Yi Zhang, Zhou Qin, Jingxuan Liang, Fanjiang Ou, Zepeng Zhang, and Shuyang Wen:** investigation, data curation. **Chunhua Ren:** supervision, methodology. **Xiao Jiang, Peng Luo, and Aifen Yan:** investigation, validation. **Haipeng Qin:** investigation, validation. **Chaoqun Hu and Ting Chen:** conceptualization, supervision, project administration, funding acquisition, writing – review and editing.

## Funding

This study was funded by the Guangdong Province Project (Grants 2024A1515010899 and 2023B1212060047), the Research on Breeding Technology of Candidate Species for Guangdong Modern Marine Ranching (Grant 2025‐MRB‐00‐001), and the National Key Research and Development Program of China (Grant 2022YFD2401301).

## Ethics Statement

The animal experiments were conducted in accordance with the guidelines and approval of the Animal Ethics Committee of the South China Sea Institute of Oceanology, Chinese Academy of Sciences.

## Consent

The authors have nothing to report.

## Conflicts of Interest

The authors declare no conflicts of interest.

## Supporting Information

Additional supporting information can be found online in the Supporting Information section.

## Supporting information


**Supporting Information 1** Table S1: Summary statistics of transcriptome sequencing data for each sample. Table S2: Principal coordinates analysis (PCoA) coordinates of transcriptomic profiles for all samples. Table S3: Summary of differentially expressed genes (DEGs) identified in pairwise comparisons among experimental groups. Table S4: Gene expression matrix of transcriptomic profiles across experimental groups. Table S5: KEGG pathway enrichment analysis of differentially expressed genes (DEGs). Table S6: Numbers of taxonomic units detected at each taxonomic rank in microbial sequencing data for each sample. Table S7: Phylum‐level taxonomic composition of microbial communities across all samples. Table S8: Class‐level taxonomic composition of microbial communities across all samples. Table S9: Species‐level taxonomic composition of microbial communities across all samples.

**Supporting Information 2** Figure S1: GO enrichment analysis of DEGs in the comparison between GA1 and Con. Figure S2: GO enrichment analysis of DEGs in the comparison between GA2 and Con.

## Data Availability

The raw RNA‐seq reads generated in this study have been deposited in the NCBI Sequence Read Archive (SRA) under BioProject accession PRJNA1418901.
