# Supplementary figures and images for: Effects of Gallic Acid Supplementation on Intestinal Function and Gut Microbial Community Structure in Holothuria leucospilota
Source: Aquac Nutr. 2026 Jul 24;2026:5350878. doi: 10.1155/anu/5350878 (PMC13400708; doi:10.1155/anu/5350878)

Description

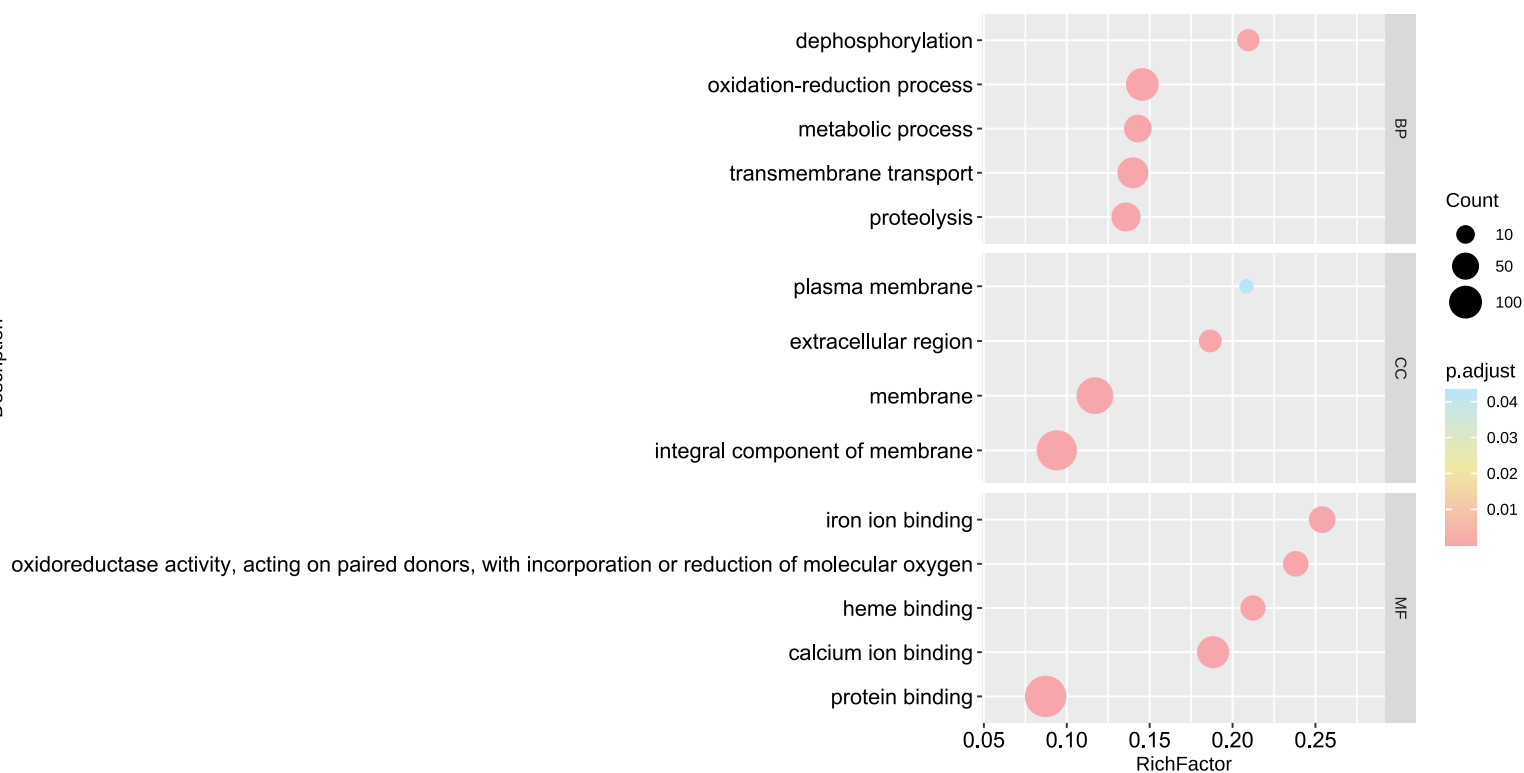

Description

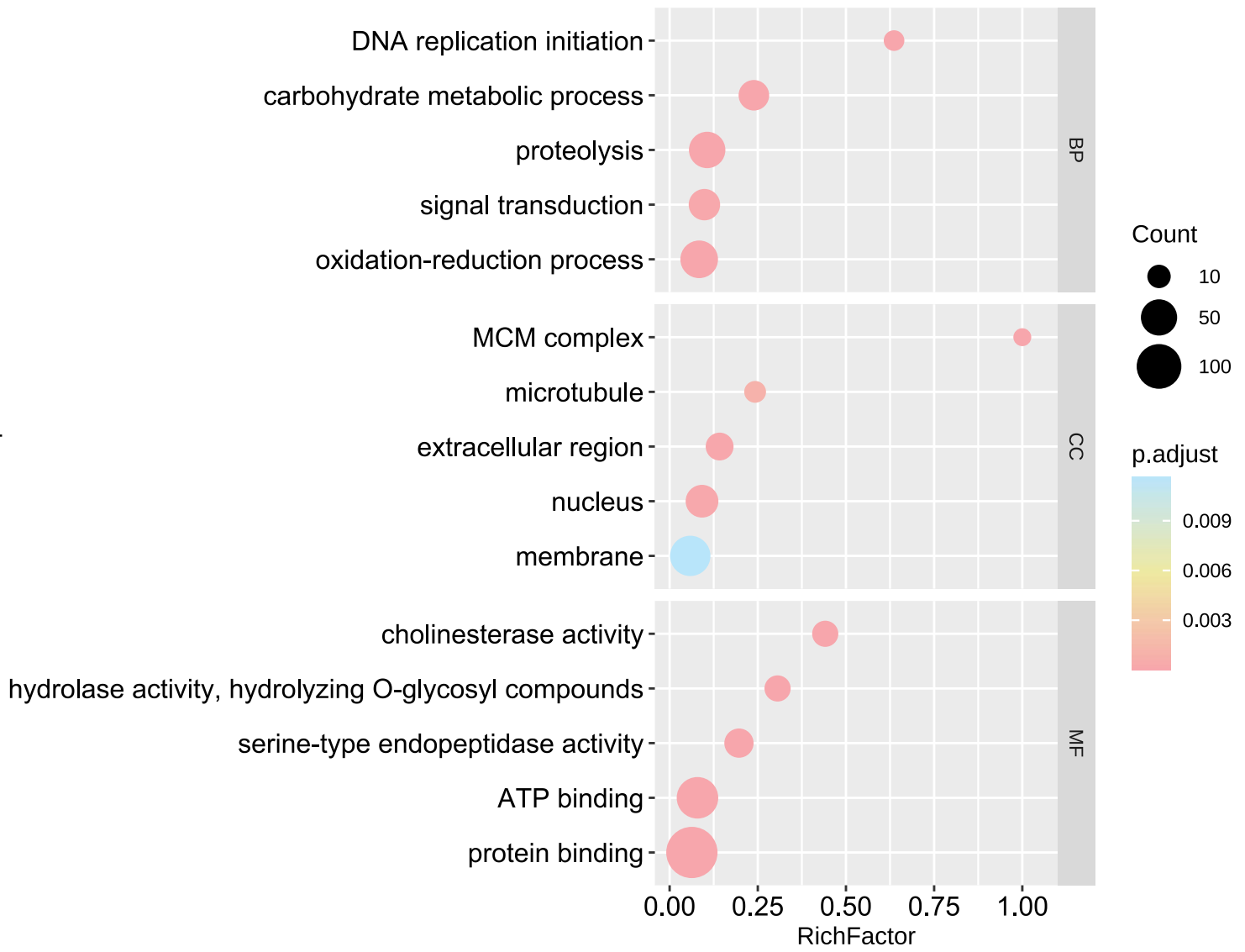

Supplement: Supplementary file 2 — Supporting Information 2 Figure S1: GO enrichment analysis of DEGs in the comparison between GA1 and Con. Figure S2: GO enrichment analysis of DEGs in the comparison between GA2 and Con. [file ANU-2026-5350878-s002.pdf]
